# Programmed cell death pathways as targets for developing antifilarial drugs: Lessons from the recent findings

**DOI:** 10.1111/jcmm.17913

**Published:** 2023-08-22

**Authors:** Nabarun Chandra Das, Pritha Chakraborty, Samapika Nandy, Abhijit Dey, Tabarak Malik, Suprabhat Mukherjee

**Affiliations:** ^1^ Integrative Biochemistry & Immunology Laboratory, Department of Animal Science Kazi Nazrul University Asansol India; ^2^ Department of Life Science Presidency University Kolkata India; ^3^ School of Pharmacy Graphic Era Hill University Dehradun India; ^4^ Institute of Health Jimma University Jimma Ethiopia

**Keywords:** apoptosis, drug development, filariasis, oxidative stress, ROS

## Abstract

More than half a century has passed since the introduction of the National Filariasis Control Program; however, as of 2023, lymphatic filariasis (LF) still prevails globally, particularly in the tropical and subtropical regions, posing a substantial challenge to the objective of worldwide elimination. LF is affecting human beings and its economically important livestock leading to a crucial contributor to morbidities and disabilities. The current scenario has been blowing up alarms of attention to develop potent therapeutics and strategies having efficiency against the adult stage of filarial nematodes. In this context, the exploration of a suitable drug target that ensures lethality to macro and microfilariae is now our first goal to achieve. Apoptosis has been the potential target across all three stages of filarial nematodes viz. oocytes, microfilariae (mf) and adults resulting in filarial death after receiving the signal from the reactive oxygen species (ROS) and executed through intrinsic and extrinsic pathways. Hence, it is considered a leading target for developing antifilarial drugs. Herein, we have shown the efficacy of several natural and synthetic compounds/nanoformulations in triggering the apoptotic death of filarial parasites with little or no toxicity to the host body system.

## 1 INTRODUCTION

Parasitic diseases spreading by helminths worldwide are rising as a serious concern for both humans and domestic animals mostly in underprivileged and unhygienic conditions. Some of these filarial parasites target the lymphatic system of the host resulting in lymphedema, lymphangitis and hydrocele affecting the arms, legs, breasts or external genitalia, leading to lymphatic filariasis (LF).^1^, ^2^, ^3^ LF is caused by *Wuchereria bancrofti*, *Brugia malayi* and *Brugia timori* which complete their life cycle within two hosts.^4^, ^5^ They are transmitted via the mosquitoes like *Culex quinquefasciatus*, *Mansonia*, *Anopheles* and *Aedes* during the nighttime as they are known to follow a circadian rhythm in which mf resides in the arterioles of the lung during the daytime, while at night they migrate through peripheral blood vessels of the host body.^6^, ^7^ The transmission process of LF involves the mosquito acquiring microfilariae (mf) from an infected host during a blood meal, followed by larval development to become infective.^2^, ^8^, ^9^ When the mosquito takes another blood meal from a healthy host, the infective larvae are transmitted to the new host.^10^ These larvae then migrate through the circulatory system, eventually reaching the lymphatic vessels where they undergo further development. Over a period of 6–12 months, they grow into adult males, measuring approximately 40 mm, and viviparous females, reaching sizes of up to 120 mm. During their lifespan of 4–6 years, successful mating occurs, resulting in the production of millions of mf.^11^ *W. bancrofti* also referred to as bancroftian filariasis accounts for 90% of the disease burden and is prevalent in tropical regions around the globe. On the other hand, Malayan filariasis is confined to Southeast Asia.^6^

Other forms of filariasis include subcutaneous and serous cavity filariasis. Subcutaneous filariasis is caused by *Onchocerca volvulus*, which primarily affects the eyes and leads to a condition called Onchocerciasis, commonly known as river blindness.^12^ On the other hand, *Loa loa* localizes in the conjunctiva of the eye, causing Loiasis.^13^ The prevalence of Loiasis is concentrated in the rainforest areas of West and Central Africa, with its transmission being reliant on Chrysops flies.^14^ In contrast, *O. volvulus* is transmitted by black flies, *Simulium* sp.^15^

According to the data published by the World Health Organization (WHO) in 1995, onchocerciasis was predominantly found in African countries, as well as certain American and Asian countries.^16^, ^17^ It affected over 17.7 million individuals, out of which 270,000 were blind and 500,000 had severe visual impairment.^18^ To encounter this scenario, various elimination programs were launched very soon after the consequences of onchocerciasis in the countries of America and Africa like, the Onchocerciasis Control Programme (OCP), the Onchocercia Elimination Programme in America (OPEA) and the African Programme for Onchocerciasis Control (APOC). Following the implementation of the elimination program, the Global Burden of Disease Study conducted in 2017 estimated that out of 20.9 million individuals infected with filariasis worldwide, approximately 14.6 million suffered from skin diseases, and 1.15 million were affected by blindness.^12^, ^19^ Thus, a new program namely the Expanded Special Project for Elimination of Neglected Tropical Disease (ESPEN) was launched in the African region in May 2016 through the cumulative efforts of WHO and the Regional Office for Africa (AFRO).^20^

Serous cavity filariasis is caused by *Mansonella perstans*, *Mansonella ozzardi* and *Mansonella streptocerca* and it is predominantly characterized by being asymptomatic.^21^ The symptoms exhibited are non‐specific, including pruritus, urticaria, arthralgia, abdominal pain and fatigue. As a result, this disease has been overlooked and neglected, lacking preventive or control programs for the affected population in endemic areas.^22^ Infections are prevalent in Sub‐Saharan Africa, Central and South America and the Caribbean, among which Africa alone reports approximately 114 million cases of *M. perstans* infection.^23^ Mf of *M. perstans* and *M. ozzardi* can be found circulating in the peripheral blood, while *M. streptocerca* is restricted to the skin. The length of these mf ranges from 160 to 240 μm, varying by species.^21^, ^22^ The absence of evidence regarding morbidity, mortality and clinical trials, coupled with contradictory transmission data, has contributed to a scenario where the government has not taken significant action to address this endemicity.

This study focuses on the current global scenario of LF and highlights the advancements made in developing therapeutics to alleviate the impact. LF stands as one of the oldest and most debilitating neglected tropical diseases (NTDs), posing a significant obstacle to the socio‐economic development of affected countries. According to the WHO, in the year 2000, LF infection was found in more than 120 million individuals across 72 countries in the tropical and subtropical regions of Asia, Africa, the Pacific, parts of the Caribbean, and South America.^24^, ^25^ However, despite efforts, there are still 51.4 million individuals suffering from LF in 44 countries.^26^ As of 2021, a staggering 882 million individuals from LF‐endemic zones remained at risk of LF, emphasizing the continued need for preventive therapy.^27^ The study estimated approximately 487 million people in India, accounting for 55.27% of the global population at risk, are under the LF threat.^26^

WHO has taken the responsibility of managing filariasis cases worldwide and has recommended a preventive treatment strategy called mass drug administration (MDA).^28^, ^29^ After the implementation of the National Filariasis Control Program in 1962, the members of the World Health Assembly developed a national plan to combat these neglected diseases in 1997. Subsequently, WHO initiated the Global Programme to Eliminate Lymphatic Filariasis (GPELF) with the goal of eliminating LF as a public health problem by the year 2020. The program established two key objectives. The first objective is to disrupt LF transmission by implementing MDA to the entire population residing in endemic areas. This involves the administration of a single annual dose of diethylcarbamazine (DEC) or a combination of ivermectin (IVM) and albendazole (ALB). The second objective aims to improve the living conditions of the affected individuals by providing essential amenities such as hygiene, skin care and increased access to surgery for men with hydrocele, in order to alleviate their sufferings.^27^, ^29^ Additionally, controlling measures targeting the vector population were also implemented. In 2000, the Global Alliance to Eliminate Lymphatic Filariasis (GAELF) was established to support GPELF by facilitating advocacy, coordinating partnerships and mobilizing the resources.^30^ Over the period of 20 years following the national plan, results are not satisfactory. There are very few countries with little success achieved as per the desired level. According to the data published by WHO in 2021, approximately, 9 billion treatments were given to more than 935 million people for a period of 21 years but there are still 44 countries worldwide including India where LF is considered a serious public health problem and the MDA program remained in ongoing status.^27^ So, an effective and compliant strategy or drug candidate or drug target must be introduced now to overcome the detrimental disease as soon as possible.

Several reports of fatalities^31^ and visual loss^32^ in onchocerciasis patients during oral DEC treatment draw a line of limited application. Intriguingly, the study of Taylor et al. and Hutchinson et al.^33^, ^34^, ^35^ scrutinized that the oral administration of DEC is less severe than the application of DEC lotion throughout the body since it experiences severe pruritus, oedematous papular rash and sometimes vascular eruption along with fever, dizziness, headache and lymphadenitis. Nowadays, this alternative use of DEC as cream has become a common tool for diagnosing onchocerciasis, as it causes the appearance of pruritic papules in the death of mf.^35^, ^36^ However, Food and Drug Administration (FDA) and Centers for Disease Control and Prevention (CDC) have authorized the oral use of DEC, along with IVM, and ALB as anti‐filarial drugs. Currently, these are the only options to treat LF and are widely used despite their significant limitations, increasing resistance and high cost.

Due to the compromised efficacy of the existing therapeutic options caused by side effects and limitations, there is an urgent requirement for the development of a new drug that can effectively target all stages of filarial nematode infections. Reports from Mexico indicate the reappearance of *O. volvulus* mf due to their resistance to DEC.^37^, ^38^ Studies conducted by Awadzi et al., Nana‐Djeunga et al. and Osei‐Atweneboana et al.^39^, ^40^, ^41^, ^42^, ^43^ in the endemic communities in Ghana and Cameroon provided evidence of IVM‐resistant *O. volvulus*. Several studies have explored the prevalence of resistance to benzimidazole, levamisole, IVM and moxidectin in *Haemonchus contortus*, *Trichostrongylus colubriformis* and *Cooperia* spp., which are nematodes affecting sheep, goat and cattle respectively (as reviewed in Howell et al.^44^). Additionally, the emergence of resistance to ML in *Dirofilaria immitis* was first reported by Hampshire in 2005.^45^ The rapid rise of anthelmintic resistance in the field of veterinary has become a matter of concern for livestock affecting the human populations as well. Resistance to benzimidazoles was found to be associated with Tyr 200 substitution in *W. bancrofti* when treated in combination with IVM^46^ although no history of resistance was found when treated alone.^47^

Multiple research reports suggest that MDA may contribute to the resurgence of the infection, but it cannot be attributed as the sole cause for this upsurge.^48^, ^49^ With the current scenario, there is a potential risk that other nematodes may develop resistance to these drugs in the near future, leading to a reemergence of the disease that could affect the population on a larger scale, particularly impacting the health of adults and children in the endemic areas.^50^ Compared to subcutaneous and serous cavity filariasis, LF is considered as the most debilitating one. Recent epidemiological data highlights that a substantial population (519 million) in South‐East Asia remains at a high risk of LF despite the extensive implementation of MDA.^26^ The lack of effectiveness of MDA is also evident in India, where 174 LF‐endemic districts have been identified.^51^, ^52^ Therefore, it is crucial to prioritize the development of new therapeutic drugs that are resistant‐free, affordable and effective, while simultaneously identifying new targets to eradicate both mf and adult parasites, including the assurance of safety of the human host. Exploring the immunological defence of the host and targeting apoptosis would be effective options to fulfil the need. Several studies have documented the use of phytochemicals, plant extracts, synthetic compounds, and nanoparticles for treating filarial parasites through inducing apoptosis.^53^, ^54^, ^55^ Herein, this article enumerates the present status of the efficacy of the antifilarial medications and their potency in exploiting apoptosis as a target for the therapeutic intervention of LF.

## 2 CURRENT STATUS OF ANTIFILARIAL DRUGS AND DRUG TARGETS FOR TREATING LYMPHATIC FILARIASIS

Several drugs have been developed throughout the decades to treat filariasis but their action kinetics are different providing different targets to explore. The following section provides the data about the anti‐filarial chemotherapeutics which have been characterized as per their targets.

### 2.1 Cytoskeleton disruptors

ALB, mebendazole, flubendazole, oxibendazole and fenbendazole all the active derivatives of benzimidazole (product of benzene and imidazole) are well known for anthelmintic action but have a history of slow absorption through gastrointestinal tract and teratogenicity.^56^ Mebendazole and flubendazole have a strong inhibition role in microtubule formation which acts as an inducer to the loss of cytoplasmic microtubules of the tegumental and intestinal cells of nematodes, followed by reduced glucose uptake^57^, ^58^, ^59^, ^60^ has provided evidence that anthelmintic benzimidazole competes with colchicines binding site to make bonding with nematode β‐tubulin.

#### 2.1.1 Ion channel blockers

Glutamate‐gated chloride channels (GluCls) are restricted only to invertebrates hence it can be a potential target to work against filariasis.^61^ IVM is currently in use which is a chemically modified naturally produced avermectin B1 which is a macrocyclic lactone (ML) showing activity against a broad spectrum of parasitic nematodes after oral or parenteral administration. It signals to open GluCl channels in maximum number and induces paralysis of pharyngeal pumping. In *Caenorhabditis elegans*, the *glc‐1* gene partly encoding the GluCl α subunit is the major target of IVM.^62^, ^63^ Concurrently, the presence of *avr‐14* from the uterine wall of female *B. malayi* and the embryonic stage of *B. malayi* mf explain the reason behind the suppression of mf production by female worms after the successful avermectin treatment.^64^, ^65^ However, a study by Dent et al.^66^ sighted that mutation of three genes from *C. elegans glc‐1, avr‐15* and *avr‐14* simultaneously endue resistance to IVM, whereas no or little resistance when two of them were mutated. Moxidectin and milbemycin oxime belong to the milbemycin family, which is consanguineous to avermectin and has expanded the area of affectivity of MLS as ion channel blockers.^67^ Piperazine is a GABA agonist acting on the ligand‐gated Cl^−^ channels on the synaptic and extra‐synaptic membrane of nematode muscles, leading to hyper‐polarization of membrane potential while keeping the Cl^−^ channels in an open state resulting in a malfunctioned sinusoidal movement of nematode which is also called flaccid paralysis.^68^ Nicotinic agonist compounds like Imidazothiazoles (levamisole, butamisole), tetrahydro pyrimidone (pyrantel, morantel, oxantel), quaternary ammonium salts (bephenium, thenium), pyrimidines (methylidene), tribendimidine and spiroindoles depolarizes the membrane potential with increased spike frequency to induce contraction on nematode muscle.^68^, ^69^, ^70^ Though the study of Colquhoun and Sakmann in 1985 minutely explained levamisole, pyrantel, morantel, and oxantel are organic cations of large size and trying to pass through the nicotinic ion channels from the outside can block the selective filter and produce spastic paralysis on nematode.^71^, ^72^ Amino‐acetonitrile derivatives (AAD) like monepantel block nicotinic acetylcholine receptors,^73^, ^74^, ^75^ whereas cyclic depsipeptides are responsible for their target to potassium channels and latrophilin receptors.^73^

### 2.2 Metabolic inhibitors

Lactate produced from carbohydrate metabolism is the major energy source for the filarial parasite in which DEC, antimonials, suramin, benzimidazoles and levamisole targets different enzymes on different steps of carbohydrate metabolism and reduce the metabolism to yield a poor amount of energy. Inhibiting the activity of phosphoenolpyruvate carboxykinase, fumarate reductase and succinate dehydrogenase by DEC alters the rate of carbohydrate metabolism^76^ while suramin inactivates lactate dehydrogenase, malate dehydrogenase,^77^ and malic enzyme^78^ in *O. volvulus* that ceases tri‐carboxylic acid cycle. According to Saz and Dunbar,^79^ a very high dose of antimonial stibophen is required to block the activity of phosphofructokinase (PFK) in filariae *Litomosoides carinii*, *Acanthocheilonema vitae* and *Brugia pahangi* by targetting glycolysis. Again DEC, benzimidazoles, and levamisole confirmed their role as glucose metabolic inhibitors by altering glucose uptake, inhibiting glucose transport, and declining glucose utilization by shifting towards homolactate fermentation respectively.^80^ Interconversion of folate derivatives in nematode is obstructed by the action of DEC and suramin on the enzymes catalysed by the whole folate metabolic process; catastrophic to nematode due to retarded purine synthesis. In particular, suramin, a highly toxic drug, catalysing the inhibition of dihydrofolate reductase (DHFR) in *O. volvulus* and NADP‐dependent 10‐formyl FH_4_‐dehydrogenase in *B. pahangi* ^80^, ^81^ but DEC mediates several inhibitions to different enzymes viz., serine hydroxymethyltransferase, 5, 10 methylene FH_4_ reductase, methylene FH_4_ dehydrogenase, 5 formyls FH_4_ cycloligase, 10 formyls FH_4_ dehydrogenase and glutamate formiminotransferase in folate metabolic pathway.^80^, ^82^ Arsenical melarsen oxide with its toxic and idiosyncratic properties showed responsibility in depleting GSH levels by inhibiting the enzyme glutathione reductase (GR) in cattle filariae *Setaria digitata*, *Onchocerca gutturosa* ^80^, ^83^ and in *L. carinii* filariid of cotton rat whereas also show little susceptibility to human erythrocyte GR.^84^ Another metabolic inhibitor Isothiocyanate amoscanate has effectivity against *L. carinii* and *B. pahangi* as micro and macrofilaricidal in inhibition glucose uptake and incorporation in glycogen.^80^, ^81^, ^82^, ^83^, ^84^, ^85^ In addition, mevinolin, an inhibitor of HMG‐CoA reductase significantly blocks the synthesis of geranyl geraniol, ubiquinone and dolichol in filariid *B. pahangi*.^80^, ^86^

### 2.3 Arachidonate metabolism blockers

Antifilarial drugs that target arachidonate metabolism have been investigated. DEC has emerged as a notable nematocide due to its ability to suppress the metabolism of arachidonic acid. It achieves this by blocking cyclo‐oxygenase (COX), which hinders the production of prostaglandin and prostacyclin—potent inflammatory responses.^68^, ^87^ DEC also blocks leukotriene synthase, a significant mediator of immune response, converting it into leukotriene.^68^, ^88^, ^89^ Razin et al.^90^ have reported that the induction of DEC does not inhibit 5‐lipooxygenase.

### 2.4 Oxidative stress inducers

Antifilarial drug DEC can block arachidonic acid metabolism, whereas aspirin non‐steroidal anti‐inflammatory drugs inhibit prostaglandin H synthase. According to Singh et al.,^91^ the combined effect of both drugs increases the level of nitric oxide (NO) and decreases the GSH and peroxidase levels to induce oxidative stress.

### 2.5 Anti‐Wolbachial therapy

The Discovery of *Wolbachia* endosymbiosis with several filarial nematodes gave new hope to invent novel strategies in the field of anthelmintic therapies.^92^ Doxycycline has an antibacterial property with the potential to reduce the plasma level of vascular endothelial growth factor‐A (VEGF‐A) which is a key regulator in hydrocele development of lymphatic filarial patients.^93^ According to Sanprasert et al.,^94^ combined therapy of doxycycline and DEC first depletes the *Wolbachia* and then kills the parasite with the potentiality of DEC. To avoid the development of resistance to antibiotics for *Wolbachia*, rifampicin and doxycycline application had been declined to the threshold level^94^ but these antibiotics have some limitations in application to children and pregnant mothers.^95^ Though clinical trials of antibiotics like doxycycline and rifampicin either in single or in combinational use have achieved numerous successful outcomes^96^, ^97^ there are also reports of a resurgence of *W. bancrofti* after 24 months of doxycycline and rifampicin combined therapy.^98^ Thailand studies have published the data revival of infection of mf and adult bancroftian filarial after 6 months of combined therapy of doxycycline and DEC.^94^

### 2.6 Combinatorial chemotherapy

ALB, IVM and DEC are the most popular therapeutic choice since the 20th century and approved by the FDA and CDC. Depending on the complexity of the disease, these drugs are sometimes used solely and sometimes in a group of two or three in different combinations like combining DEC with aspirin and achieve the beneficial role of aspirin in the induction of oxidative stress. While combined groups such as DEC + ALB and IVM + ALB gave an idealistic opportunity to aim different targets at a single exposure and also for quick action replay, there was an expectation of no new resistance. Treating LF with co‐infection of *Wolbachia* combinational therapy of doxycycline with DEC also proved the success behind the cooperation of combined drugs and their targets. IDA is a new triple‐drug‐based treatment therapy as recommended by WHO, by combining IVM, DEC and ALB and in comparison, to the two‐drug regimen, the trial of IDA against *W. bancrofti* in Papua New Guniea showed a low success in sterilizing adult parasite for a period of only 3 years^99^ than DEC and ALB combined provides microfilaricidal activity.^100^ Other studies performed using DEC 6 mg/kg along with ALB 400 mg showed a high reduction of adult *W. bancrofti*.^101^, ^102^ Subsequent studies carried out in American Samoa starting from 2001 and spanning over a period of 6 years, involving combined MDA of ALB and DEC, demonstrated a significant reduction in the prevalence of *W. bancrofti* antigenemia. The prevalence dropped from 11.5% in 2001 to 0.95% in 2006.^103^ ALB alone is worse in comparison to IVM but combined with IVM it has shown a higher mf reduction rate along with a significant clearing ability of mf from night blood.^104^, ^105^ Makunde et al.^106^ demonstrated that IVM + ALB therapy is safe and endurable in treating bancroftian filariasis when coinfected with onchocerciasis. Report from a study of nine villages in South India of re‐emergence of *W. bancrofti* after the successful MDA with an annual single dose of IVM 400 μg/kg with DEC 6 mg/kg rejected the new theory of combinational chemotherapy in treating filariasis but the sustainable result came out when suitable vector control measure all‐night landing catch (ANLC) method had accessed against *C. quiquefasciatusis*.^107^ Interestingly, several clinical trials on *W. bancrofti*‐infected communities also showed that there were no differences in efficacy between IVM alone and combination therapy with DEC^108^ and again in between IVM alone with IVM + ALB treatment.^109^

### 2.7 Life cycle inhibitors

**FIGURE 1:**
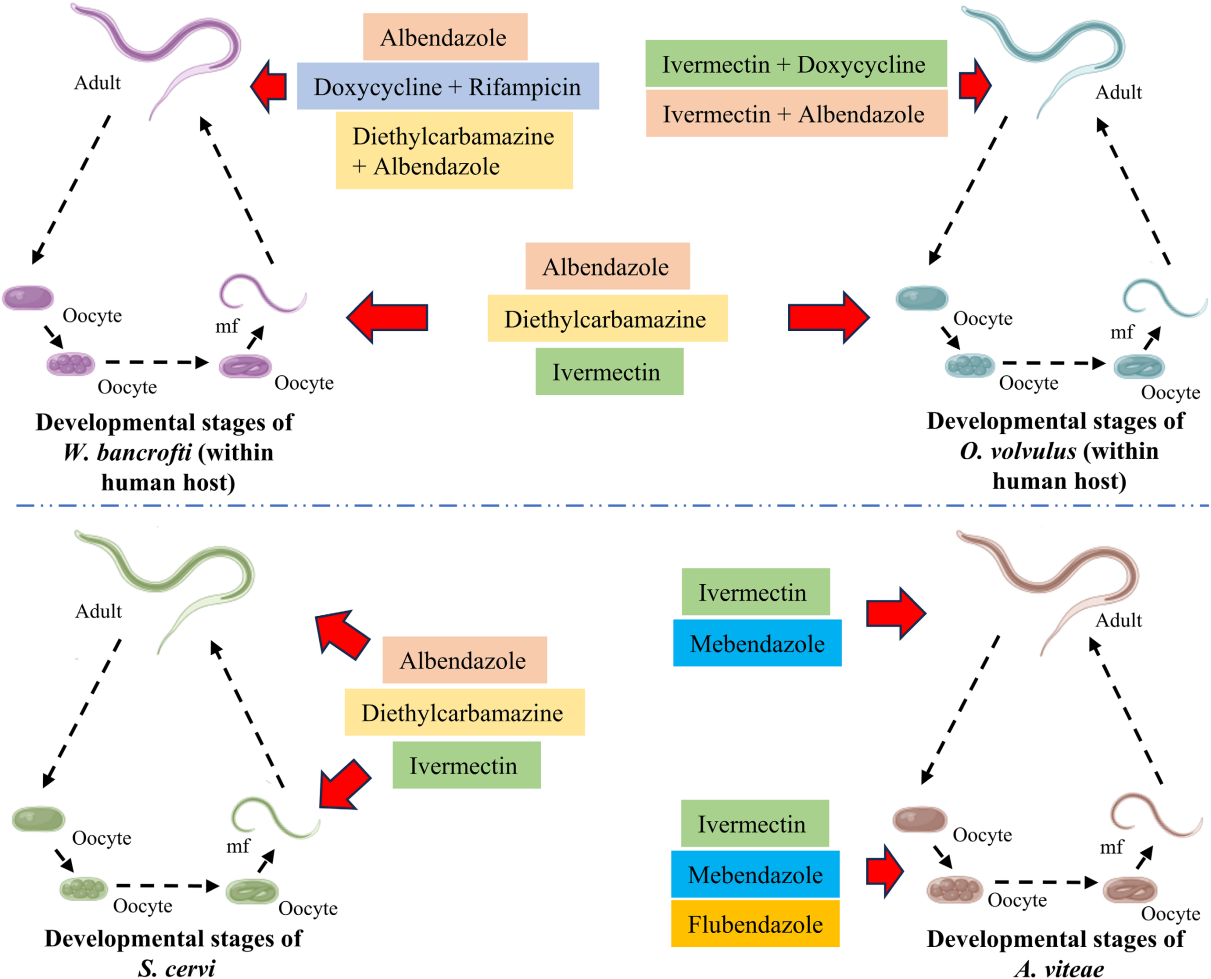
Schematic representation of the potential efficacy of different chemotherapeutics across the developmental stages of filarial parasites reside in the human host (*Wuchereria bancrofti* and *Onchocerca volvulus*), bovine host (*Setaria cervi*) and rodent host (*Acanthocheilonema viteae*).

Targeting and blocking the life cycle of the filarial parasites are crucial strategies in reducing transmission and infection burden. Analysis of numerous epidemiological and clinical trial studies has revealed that the components of MDA have limited efficacy against both mf and adult filarial parasites. Furthermore, the effectiveness of these components varies among different species causing filariasis (Figure 1). While few exhibit efficient ovicidal and microfilaricidal properties, their efficacy against adult parasites is often diminished or delayed.^110^, ^111^, ^112^, ^113^, ^114^, ^115^ Antibiotics such as doxycycline and rifampicin have demonstrated better results in eliminating human parasites that harbour *Wolbachia* as an endosymbiont.^94^, ^96^, ^97^ However, these antibiotics have been found to be ineffective against bovine or rodent parasites. Studies have also reported instances of mf and/or antigenemia reemergence months after treatment, indicating the inability of chemotherapeutic agents to fully block the life cycle of filarial parasites. Failures of drugs like DEC, ALB and IVM are not limited to filarial parasites alone, as similar reports have been observed with other parasite species as well. DEC, for instance, has shown no efficacy against schistosomiasis‐causing parasites. On the other hand, ALB, when used in combination with praziquantel and nitazode, targets larval forms of *Schistosoma mansoni* and eggs of *Schistosoma japonicum*, *Schistosoma haematobium* and *S. mansoni*.^116^, ^117^, ^118^ Interestingly, IVM has exhibited better efficacy against all the developmental stages of the parasites responsible for schistosomiasis.^119^, ^120^, ^121^

## 3 APOPTOSIS AS A POTENTIAL DRUG TARGET FOR TREATING THE FILARIAL WORMS

Experiments based on evidence have proposed the idea of exploring the natural process of innate response to eradicate infective pathogens has given us the idea to target the host natural immune response and promote the defence system to eradicate lymphatic filariasis. DEC has given evidence of inducing apoptosis in vitro and killing the parasite efficiently. Infected cells undergo programmed cell death called ‘apoptosis’ in which the infected cells or dead cells are removed by the host immune system to maintain the homeostatic balance. During the apoptotic process, the cytoskeleton gets modified resulting in membrane blebbing, chromatin condensation and degradation of DNA into smaller fragments thus resulting in a significant decrease in cell volume. H. Robert Horvitz was the first, who worked on apoptosis and developed a complete pattern of programmed cell death after thorough observation of living cells of *C. elegans*.^122^, ^123^ Apoptotic cells after the formation of apoptotic bodies are engulfed by macrophages. They never release any substances like lytic enzymes or oxidizing molecules that may induce a local inflammatory response.

### 3.1 Apoptosis in filarial worms: extrinsic and intrinsic pathways

The filaricidal actions used on the parasites have resulted in the low concentration of Superoxide dismutase (SOD), catalase and Glutathione Peroxidase (GPx) enzymes acting as a vital antioxidant defence mechanism.^124^ GSH is a sulfur‐containing protein that carries reactive electrons from the peroxide that get affected and lowers its concentration, due to filaricidal induction whereas glutathione‐S‐transferase (GST) maintains a higher concentration of ROS in the mitochondria, endoplasmic reticulum, and peroxisome thus resulting in apoptosis.^125^

**FIGURE 2:**
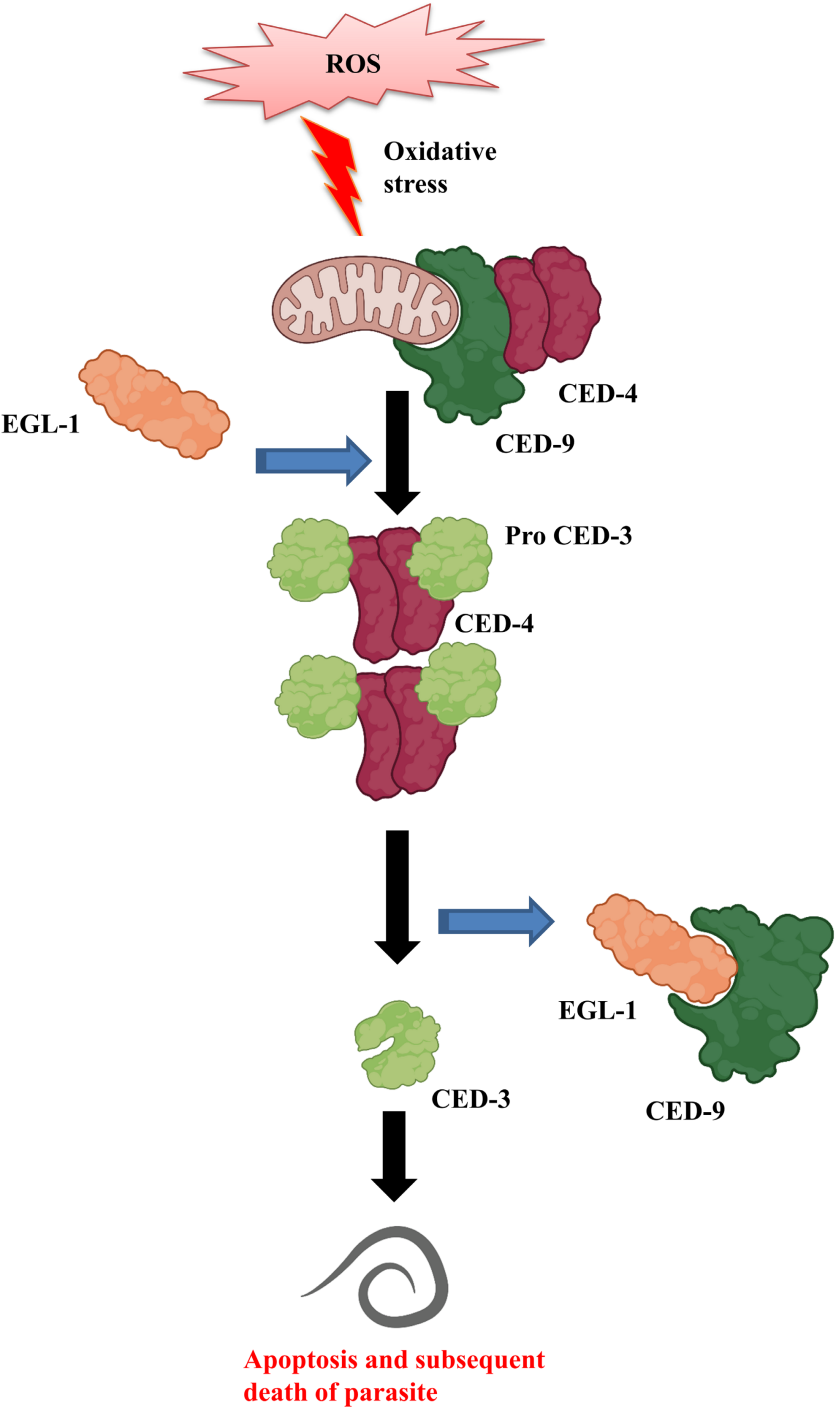
Mechanism of apoptosis in filarial parasites. ROS‐induced oxidative stress within the filarial parasites activates a major cell death pathway. EGL‐1 the apoptosis inducer disrupts the normal CED‐4–CED‐9 complex form and makes an association with CED‐9 to create a pro‐apoptotic moiety. Free CED‐4 dimers then oligomerize and make tetrameric apoptosomes with proCED‐3. Activated and cleaved CED‐3 is the ultimate protein responsible for apoptosis in filaria parasites.

Egg‐laying defective (EGL)‐1, cell death abnormal (CED)‐3, (CED)‐4 and (CED)‐9 are four essential proteins with their corresponding role as pro‐apoptotic and anti‐apoptotic proteins mediating the role in apoptosis in filarial parasites.^53^ EGL‐1 and CED‐9 proteins represent the bcl2 family, whereas CED‐4 is the nematode homologue of mammalian apoptotic protease‐activating factor‐1(Apaf‐1). During apoptosis, CED‐9 is negatively regulated by EGL‐1 and fails to show dominancy over CED‐4 and CED‐3.^122^, ^126^ In a normal filarial cell, CED‐4 dimers are sequestered with CED‐9 on the outer surface of the mitochondria and inhibit apoptosis.^127^ In time, stimulation after an apoptotic induction increased the level of EGL‐1 and made bonding with CED‐9 by the BH3 domain that in turn disrupting the CED‐4–CED‐9 complex.^128^, ^129^ After dissociation of CED‐9, two asymmetric CED‐4 dimers oligomerize to make a tetrameric apoptosome and this tetrameric structure then recruits proCED‐3 molecules^130^ followed by CED‐3 a cysteine protease becomes activated and executes apoptosis, which is nematode specific^126^, ^127^ (Figure 2). Peixoto et al.^131^ weref parasite by among the pioneers who provided molecular evidence of apoptosis in the filarial parasite *W. bancrofti*. Further studies conducted by Landmann et al.^126^ and Roy et al.^132^ demonstrated that the CED pathway operates the induction, progression and execution of apoptosis in the filarids. The signalling molecules of CED pathway are analogous to the pathway operative in the free‐living nematode *C. elegans*.^133^, ^134^ Existence of the CED pathway was found to be operative in both wolbachial (*W. bancrofti* and *B. malayi*) and non‐wolbachial (*Setaria cervi* and *S. digitata*) filarial parasites^53^, ^126^, ^132^, ^135^ that indicated this pathway to be a ubiquitous drug target.

**FIGURE 3:**
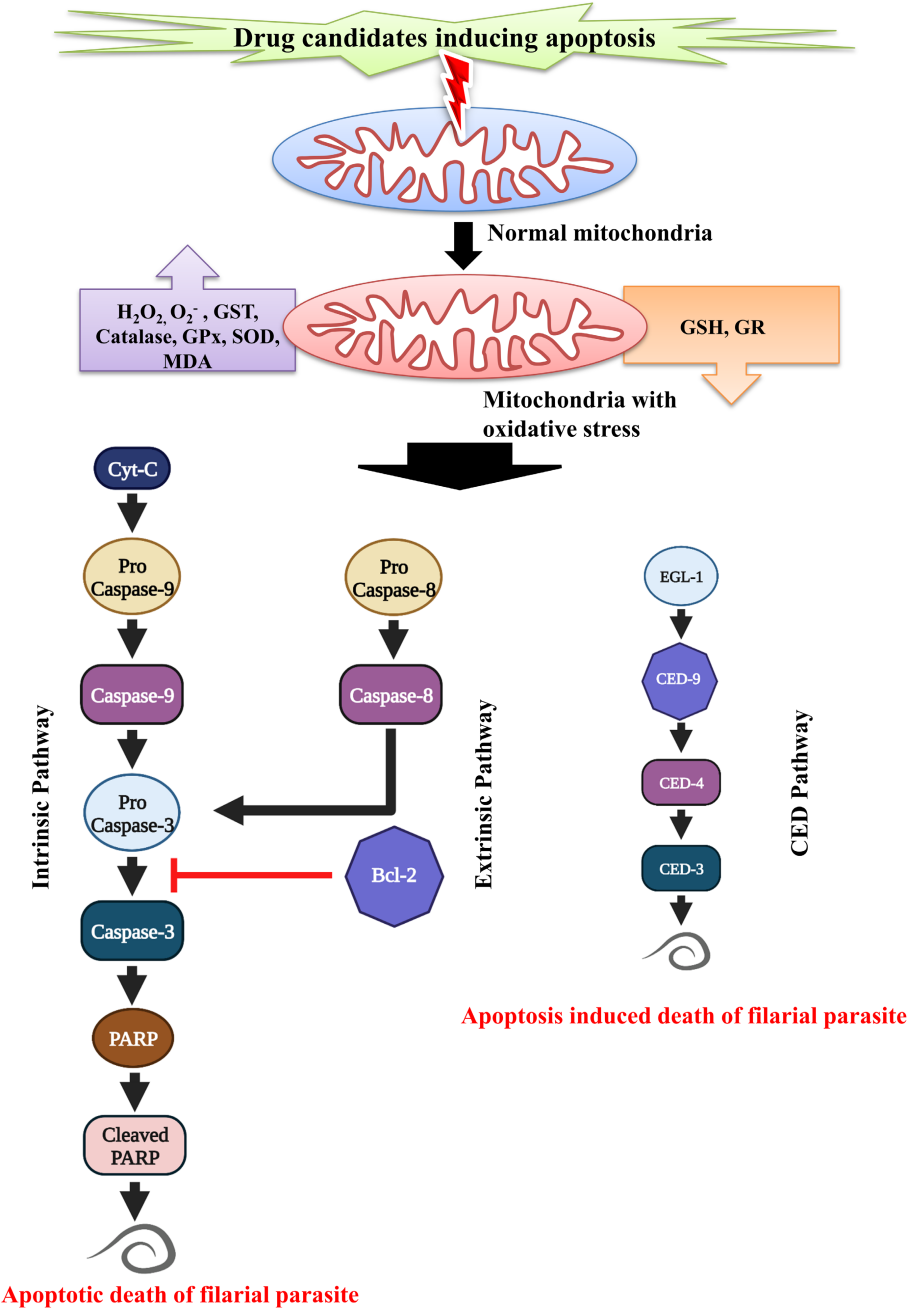
Apoptosis as a target for developing antifilarial drugs. Apoptosis‐inducing drugs primarily raise oxidative stress inside the parasite body and subsequently activate intrinsic, extrinsic as well as filarial conserved apoptosis pathways, EGL‐1/CED‐9/CED‐4/CED‐3 pathway.

Studies on filaricidal‐treated parasites by Mukherjee et al.^53^, ^125^, ^136^ revealed a rich level of cysteine protease family protein, called caspase, directing a new path which is suitable for filarial apoptosis by activating other inactive proforms by making a cleavage at a specific sequence next to aspartate. The involvement of caspase protein cas‐9, cas‐8 and cas‐3, cytochrome c and poly (ADP‐ribose) polymerase (PARP) in the nematode^125^, ^136^ indicates both intrinsic and extrinsic pathways are induced in apoptosis. In conclusion, the nematode‐specific CED pathway and the newly arise caspase pathway both have fruitfulness in executing apoptosis in the filarial parasite (Figure 3).

## 4 APOPTOSIS INDUCERS AS ANTIFILARIAL AGENTS

Natural and synthetic compounds have been considered an alternative over a couple of years since they have shown potential in killing the adult filariid by inducing apoptosis. Apoptotic target killing model has been developed in bovine parasite *S. cervi* ^137^, ^138^ and *S. digitata* ^139^, ^140^ due to their biochemical, immunological and morphological resemblance to *W. bancrofti* and brugian parasites.

### 4.1 Anthelmintic drugs

Apart from the most promising targets, MDA‐approved anthelmintic drugs also have some inductive properties to execute apoptosis in filarial parasites. Among the drugs, DEC has a crucial role as a metabolic inhibitor. According to Peixoto et al., Shakya et al. and Singh et al.,^91^, ^131^, ^141^ DEC when used as a single drug or in combination with aspirin illustrates a decisive role in mediating apoptosis. Microtubule assembly preventer ALB and *Wolbachia* depletory antibiotics like rifampicin and doxycycline when administered have shown tunnel‐positive results along with the presence of fragmented DNA and condensed chromatin proving the involvement of apoptosis in the killing of the parasite.^142^, ^143^ In animals and in vitro observations of tetracycline on *B. malayi* provide favourable information, *Wolbachia* depletion is a key to induce apoptosis to filariid.^126^

### 4.2 Phytochemicals

RT‐PCR and ROS measurement study revealed that Hydroxycinnamic acid; a phenolic compound isolated as ferulic acid from *Hibiscus mutabilis*, has a potent antifilarial effect in ROS generation and has significantly decreased the expression of CED‐9 that later, in turn, active CED‐3 to progress apoptosis in adult as well as mf in bovine filariid *S. cervi*.^144^ The tunnel positive study of Nayek et al.^145^ showed the advantageous role of the anti‐carcinogen *Curcuma longa* derived curcuminoid, curcumin to deplete the parasitic GST level and improve the ROS generation that induces apoptosis in micro as well as in macrofilariid. Apart from anti‐malarial, anti‐leishmanial, anti‐trypanosomal and anti‐nematicidal properties, ursolic acid is a pentacyclic triterpenoid carboxylic acid from *Nyctanthes arbortristis* leaves, also having efficient anti‐filarial activities in inducing CED mediating apoptosis to mf of *S. cervi* and *W. bancrofti* as well as to adult *S. cervi*.^133^ Azadirachtin is a tetranortriterpenoid phytocompound extracted from *Azadirachta indica*, elevates the level of superoxide anion and develops oxidative stress that finally leads to apoptotic death of *S.cervi*.^146^ MTT results from the study of Mukherjee et al.^146^ showed that Azadirachtin causes a significant reduction in worm viability in both mf and adult parasites. Again, Hoechst and Tunnel staining on oleanolic acid‐treated *S. digitata* provide significant evidence of chromatin condensation and apoptotic body formation among all developmental stages of the parasite.^135^

### 4.3 Plant extract

Methanolic leaf extracts of *Aegle marmelos*, commonly known as beal, are rich in polyphenol which is sufficient to generate oxidative stress at a high concentration and is considered a pro‐oxidative and pro‐apoptotic agent to microfilariae parasites.^147^ Mf of *B. malayi* treated with a high dose of methanolic root extract of *Vitex negundo* (nirgundi) loses their motility only about 50% while in combination with H_2_O_2_ achievingfull motility.^147^ According to Sahare et al.,^148^ the root extract of *V. negundo* contains saponin which is apoptotic in nature. *Butea monosperma* is well known for its medicinal value as used to treat various ailments in ‘Ayurveda’, commonly called the flame of the forest or palasa have antimicrofilariae activity against *B. malayi*.^149^ Evidence of lipid peroxidation in the *B. monosperma* leave extract used to treat microfilaria can induce oxidative stress along with the presence of saponin in the extract has validated the relation between oxidative stress with apoptosis.^149^, ^150^ *Cajanus scarabaeoides* commonly known as showy pigeonpea has diverse therapeutic value in treating anaemia, smallpox, dysentery, cholera, gonorrhoea and rinderpest as an ethnomedicine. Among the chloroformic, ethanolic, ethyl acetate and petroleum etheric extraction, ethanolic extracts derived from the stem part of *C. scrabaeoides* are polyphenol enriched and show the most feasible effect when treated against oocytes, mf and adults *S. cervi* in activating ROS‐dependent CED pathway of apoptosis.^54^ Neem tree belongs to meliaceae family used in traditional medicine for over two millennia by Siddha and Ayurvedic practitioners as anthelmintic, antifungal, antidiabetic, antibacterial, antiviral, contraceptive and sedative. TUNEL‐positive apoptotic nuclei in mf and adult *S. cervi* suggest ethanolic extraction from leaves of *A. indica* (EEA) exerts an antifilarial role in mediating apoptosis in *S. cervi*.^55^ *D. immitis*, heartworm of dogs is also an apoptotic target for EEA in which 100 μg ml^−1^ dose EEA can alter the external morphology of the mf with compressed sheath, breakages and holes across the body.^134^ Novel antifilarial agent *n*‐butanol extract (NBE) of *Diospyros perigrena* stem bark proficient in generating *in vitro* efficacy against mf and adult form of *S. cervi* by upregulating pro‐apoptotic proteins and subsequently downregulating antiapoptotic proteins.^151^ Analysis of tissue damage and hallmark features of apoptosis in treated *S. digitata* demonstrate rhizome extract of *Curcuma zedoaria* is significant to induce proapoptotic proteins thus the extract is potent as micro and macrofilaricidal.^152^

### 4.4 Synthetic compounds

Synthetic cyclic sulfonamide, sultam, sultone and cyclic sulfonates are biologically very active compounds exhibiting antiviral, antifungal and antiparasitic activities.^153^ Among these, quinolone‐fused cyclic sulfonamide (4,7‐dimethyl‐3,4,7,8‐tetrahydro‐3λ6‐[1,2]thiazino[4,3‐*f*]quinoline‐3,3,8‐trione) 8 L is a hybrid compound synthesized by fusion of coumarin and quinolone and is imperative to accumulate ROS that increases oxidative stress and later signals for apoptosis by triggering both extrinsic and intrinsic pathways against all the developmental stages of *S. cervi*. Moreover, 8 L is also beneficial against *Wolbachia* infection.^53^ More precisely, the activity of 8 L is more efficacious than IVM along with its non‐toxic nature to mammalian hosts that target apoptosis makes it the most promising drug candidate among all filaricidal compound. According to A. Gucchait et al.,^154^ compound 4a among the series of synthesized carbamo(dithioperoxo)thioate derivatives deliberate as most acceptable in inducing apoptosis to the oocyte, mf and adult stage of *S. cervi* after the flow cytometry analysis of oocyte and mf, and western blotting of apoptotic protein of adults. The involvement of apoptotic mediator caspase 3, caspase 8, caspase 9, cytochrome *c* and PARP along with filarial‐specific CED proteins provide evidence that the trans‐stilbene derivatives are efficient in triggering apoptosis in filariids.^125^ Again, trans‐stilbene derivatives along with Resveratrol (RSV) exhibit much more potency than alone RSV in mediating apoptotic death in nematode *S. cervi*.^125^ Synthesized compound C‐cinnamoyl glycoside also has much potential as an antifilarial agent as well as an apoptotic agent that leads to the permanent death of bovine parasite *S. cervi* and human‐affecting filarial parasite *W. bancrofti*.^155^ Butylated hydroxyl anisole is a well‐known food additive that is used to treate adult *S. cervi*, showing an adulticidal effect making it more precious. But compared with macrofilaricidal methyl chalcone (MC), MC takes the lead after the parasite's motility and viability test and analysis of GSH level.^156^ Derivatives of 2, 4‐diaminopyrimidine and 2, 4‐diamino‐s‐triazine are Dihydrofolate reductase inhibitors (DHFR) acting as a potent antifilarial agent with evidence from apoptosis detection ethidium bromide‐acridine orange (EB/AO) staining proves these derivatives are also responsible for apoptotic damage in *B. malayi* mf and adult.^157^ After measuring the TrxR activity in 2,4‐dinitrochlorobenzene (CDNB) treated *S. cervi* mf, it has cleared CDNB targets TrxR, imbalance the cellular redox homeostasis followed by mitochondrial membrane lipid peroxidation and protein carbonyl formation that finally initiates intrinsic pathway of apoptosis.^158^ According to Mandvikar et al.,^159^ Im10 (5‐benzylidene‐2‐imino‐3‐(4‐phenylthiazol‐2‐yl)thiazolidin‐4‐one), a thiazolidine derivative has much more potential than DEC to trigger ROS‐mediated apoptosis in treated *B. malayi* mf and adult.

### 4.5 Nanoparticles

Nanotechnology is the new future of drug discovery that uses nanoparticles as a treatment option by delivering the drugs to its targets. Several filaricidal nanoparticles have already been synthesized in the hope that they could be our future drug in treating filariasis like, chitosan functionalized gold nanoparticles which are compatible biologically with the mammalian host and have low allergenicity and biodegradability with no toxic effect. It can upregulate the production of ROS within the filarial parasite resulting in DNA fragmentation hence it is acceptable as an effective nanotherapeutic inducer against filariasis.^160^ Chitosan and *Terminalia chebula* extract‐based gold nanoparticles are synthesized by following the green method and has a remarkable anti‐filariasis effect with evidence of externalization to the membrane phosphatidylserine on treated filarial parasites after dual staining with 6‐carboxyfluorescein diacetate and annexin‐VCy3. TUNEL positive result also proves its apoptotic nature as filaricidal.^132^ Another composition of gold nanoparticles with chitosan and extract of *Piper nigrum* is very active against *S. cervi* in inducing oxidative stress, an imbalance redox and further activating pro‐apoptotic proteins.^161^ According to Roy et al.^162^ BCH (chitosan stabilized) are more significant effector than BEG (ethylene glycol stabilized) and BST (starch stabilized) silver nanoparticles (AgNP) on apoptotic cell death. When applied in high doses approx. 250 μL/mL to *S. cervi*, most of the mf and adults lose viability. Silver nanoparticles comprise biomolecule tyrosine and natural polymer starch which are highly active in ROS generation and induce apoptosis in the larval stage of filarial parasites but also in filaria causing vector *C. quinquefasiatus*.^163^ Other silver nanoparticles synthesized from funicles extract from *Acacia auriculiformis* exhibit their antifilarial effect with very little concentrated dose, that is, 50% death at only 3.58 μg/mL concentration compared with raw extract of the plant and also significant in triggering oxidative stress that finally mediates apoptosis of filariids.^164^ The study conducted by Yadav et al.^165^ indicated that the raw plant *Andrographis paniculata* extract is much weaker than the silver nanoparticle synthesized with the same plant extract in killing parasites by triggering apoptosis as well as confirming the success of nanoformulation in the treatment of filariasis.

D‐glucose and hydrazine composite silver nanoparticle is also a very potent filaricid in generating oxidative attack at a very low dose.^166^ Report of propidium iodide staining in the degradation of nuclear DNA supports the apoptotic nature of polyvinyl capped silver nanoparticles when treated against *S. cervi*.^167^ According to Zafar et al.,^168^ ALB‐copper (II) oxide nanocomposite is much more effective in establishing oxidative stress in comparison to ALB. This study also supports cost‐effective nanoformulation which can be our future therapeutic strategy to combat filariasis.

Herein, Table 1 has been specifically designed to list and provide details on all the effective natural and synthetic compounds that have been found to induce apoptosis in filaria‐causing parasites.

**TABLE 1:** Apoptosis as a potential target for developing antifilarial drugs.

Name of antifilarial agent	Chemical class	Efficacy	Targeted filarial worm	Postulated mode of action	Side effect(s)/limitation(s)	Reference(s)
*Anthelmintic drugs*						
Diethylcarbamazine (DEC)	Carbamazine	Microfilaricidal	*Wuchereria bancrofti* and *Brugia malayi*	Inhibits arachidonic acid metabolism as well as excess oxidative stress induce apoptosis	Worsen and also cause death of patients with onchocerciasis and loiasis	[131, 141]
Combination of DEC with aspirin	Carbamazine and acetoxybenzoic acid	Microfilaricidal as well as adult filaricidal	*Setaria cervi*	Inhibit prostaglandin H synthase and increase the level of NO, which in turn induce mitochondria‐leaded caspase‐mediated apoptosis	Non‐toxic; under the recommended aspirin dose	[91]
Albendazole (ALB)	Benzimidazole	Microfilaricidal and macrofilaricidal action	*S. cervi*	Tubulin disrupter and signal activation of CED pathway in the filarial worm	Non‐toxic; rate of absorption is low	[143]
Antibiotic	Tetracycline	Microfilaricidal	*B. malayi*	Excessive activation of the CED pathway after *Wolbachia* depletion	‐	[126]
Albendazole and antibiotic synergy	Benzimidazole with rifampicin	Embryocidal, microfilaricidal and most importantly macrofilaricidal	*B. malayi*	Wolbachia depletion induces apoptosis in embryonic and uterine tissues	‐	[142]
*Phytochemicals*						
Ferulic acid	Natural/synthetic phenolic compound	Ovicidal, microfilaricidal and macrofilaricidal action	*S. cervi*	Induces ROS and activates the extrinsic pathway of apoptosis in filarial worms	Non‐toxic	[144]
Curcumin	Curcuminoid phytocompound	Microfilaricidal and macrofilaricidal	*S. cervi*	Generation of ROS and elevated levels of CED‐3 and CED‐4 mediate apoptosis	Non‐toxic	[145]
Ursolic acid	Pentacyclic triterpenoid phytocompound	Potential only as microfilaricidal and macrofilaricidal	*W. bancrofti* and *S. cervi*	Increased level of ROS activates proapoptotic genes to mediate apoptosis	Non‐toxic	[133]
Olenolic acid from *Dipterocarpus zeylanicus*	Triterpene saponins	Microfilaricidal and macrofilaricidal	*S. digitata*	Enhanced ROS generation leads to oxidative stress that further induces apoptosis	Non‐toxic	[135]
Azadirachtin	Tetranortriterpenoid phytocompound	Macrofilaricidal and microfilaricidal activity	*S. cervi*	ROS‐mediated activation of the CED pathway of filarial apoptosis	Non‐toxic	[146]
*Plant extracts*						
Methanolic extract of leaves of *Aegle marmelos*	Polyphenolic phytocompound	Microfilaricidal	*W. bancrofti* and *S. cervi*	Alone or in combination with diethyl carbonate trigger apoptosis in filarial parasite *Brugia malayi*	Non‐toxic	[138, 139]
Methanolic extract of the root of *Vitex negundo*	Polyphenolic phytocompound	Microfilaricidal	*B. malayi*	Apoptosis inducer; shows better efficacy in combination with diethyl carbonate	Non‐toxic	[147, 148]
*Butea monosperma* leave extract	Saponins	Microfilaricidal	*B. malayi* and *S. cervi*	Induction of membrane peroxidation and oxidative stress triggers apoptosis in *B. malayi* microfilariae	Non‐toxic	[149, 150]
Ethanolic extratcts from leaves of *Azadirachta indica* (EEA)	Polyphenolic phytocompound	Show efficacy on mf and adult filarial	*Dirofilaria immitis* and *S. cervi*	Generation of ROS leads to alteration of proapoptotic genes and finally apoptosis	Non‐toxic	[55, 134]
N‐butanol extract of *Diospyros perigrena* bark	Butanol	Microfilaricidal and adulticidal	*S. cervi*	Decreased levels of GSH and GST generate ROS that triggers apoptosis	Non‐toxic	[151]
Hexane and chloroform extract from *Curcuma zedoaria* rhizome	Phytocompound	Microfilaricidal and macrofilaricidal	*S. digitata*	Oxidative stress leads to CED and caspase‐mediated concomitant apoptosis	Non‐toxic	[152]
Ethanolic extract of *Cajanus scarabaeoides*	Polyphenolic phytocompound	Ovicidal, microfilaricidal and macroflaricidal activities	*S. cervi*	Induce ROS generation and apoptosis	Non‐toxic	[54]
*Synthetic compounds*						
Butylated hydroxyl anisole	Phenolic compound	Adulticidal	*S. cervi*	Depletion on GSH level triggers ROS and induces apoptosis	Very low‐level toxicity	[156]
Methyl chalcone	Aromatic ketone	Macrofilaricidal	*S. cervi*	Inhibition of GSH signalled oxidative stress that leads to apoptosis	‐	[156]
2,4 diaminopyrimidine and 2,4 diamino ‐s‐triazine derivatives	Biguanides and dihydrotriazine	Microfilaricidal and macrofilaricidal	*B. malayi*	Inhibition of dihydrofolatereductase triggers apoptosis	‐	[157]
CDNB (1‐chloro‐2,4‐dinitrobenzene)	Dinitrochlorobenzene	Microfilaricidal	*S. cervi*	Inhibit TrxR leads to down‐regulation of the antioxidant system. Increased ROS induces membrane lipid per oxidation that alters mitochondrial membrane permeability and mediates cytochrome *c*‐induced intrinsic apoptosis	‐	[158]
Im10 (5‐benzylidene‐2‐imino‐3‐(4‐phenylthiazol‐2‐yl)thiazolidin‐4‐one)	Thiazolidine derivatives	Microfilaricidal as well as macrofilaricidal	*B. malayi*	Oxidative effect triggers apoptosis	Non‐toxic	[159]
Benzosultam and Benzosultone	Uracil‐derived heterocyclic sulphonamide	Ovicidal, microfilaricidal and macrofilaricidal	*S. cervi*	Induce apoptosis on filarial parasites *Setaria cervi*	Non‐toxic	[153]
C‐cinnamoyl glycoside	Natural/synthetic compound	Effective over microfilariae	*W. bancrofti* and *S. cervi*	Apoptosis	Non‐toxic	[155]
Trans‐stilbene and Resveratrol derivatives	Synthetic compound	Microfilaricidal and macrofilaricidal action	*S. cervi*	ROS generation activates the CED pathway as well as extrinsic and intrinsic pathways, leading to apoptosis	Non‐toxic	[125]
8 L (4,7‐dimethyl‐3,4,7,8‐tetrahydro‐3λ6‐[1, 2]thiazino[4,3‐f]quinoline‐3,3,8‐trione)	Quinolone linked cyclic sulfonamide	High activity as ovicidal, microfilaricidal and macrofilaricidal	*S. cervi*	Induces ROS and activates both intrinsic and extrinsic pathways of apoptosis	Non‐toxic	[53]
Carbamo (dithioperoxo) thioate derivatives	Synthetic compound	Ovicidal, microfilaricidal as well as macrofilaricidal action	*S. cervi*	Induces apoptosis via the CED pathway	Non‐toxic	[154]
*Metal nanoparticles/nanocomposites*						
D‐glucose and hydrazine nanocomposite	Silver nanoparticles	Microfiaricidal	*B. malayi*	Oxidative stress‐induced apoptosis	Non‐toxic	[166]
Polyvinyl alcohol‐capped nanoparticle	Silver nanoparticle	Micro as well as macrofilaricidal	*S. cervi*	Trigger CED control apoptosis	Non‐toxic	[167]
Tyrosine‐based and chitosan‐capped silver nanoparticles	Silver nanoparticle	Microfilaricidal and macrofilaricidal action	*S. cervi*	Induces apoptosis, following the CED pathway	Non‐toxic	[162]
Funicles extract of *Acacia auriculi formis* in silver nanoparticles	Silver nanoparticle	Ovicidal, microfilaricidal and macrofilaricidal action	*S. cervi*	ROS‐mediated apoptosis	Nontoxic	[164]
Starch‐stabilized nanoparticles	Silver nanoparticle	Microfilaricidal and macrofilaricidal	*S. cervi*	ROS‐mediated apoptosis	Non‐toxic	[163]
Chitosan‐based nanoparticle	Gold nanoparticle	Microfilaricidal and macrofilaricidal activity	*S. cervi*	ROS‐mediated apoptosis with activating CED proteins	Non‐toxic	[161]
Albendazole and copper oxide nanocomposite	Copper (II) oxide nanoparticle	Microfilaricidal and macrofilaricidal	*S. cervi*	Enhanced ROS generation mediates filarial apoptosis	Lower toxicity	[168]
Chitosan with *Terminalia chebula* extract	Gold nanoparticle	Microfilaricidal for *Wuchereria bancrofti* while both micro and macrofilaricidal to *S. cervi*	*S. cervi*	Upregulation of Nrf2 elevate GST activity and reduced GSH level trigger oxidative stress that ultimately induce apoptosis	Nontoxic	[132]
Chitosan functionalized gold nanoparticle	Gold nanoparticle	Microfilaricidal and macrofilaricidal activity	*S. cervi*	Induces ROS and activates the CED pathway of apoptosis	Non‐toxic	[160]
Green nanoparticle of *Andrographis paniculata* leaf extract	Silver nanoparticle	Macrofilaricidal	*S. cervi*	Elevated ROS generate oxidative stress that finally leads to filarial apoptosis	Non‐toxic	[165]

## 5 WHY APOPTOSIS?

All the FDA‐ and CDC‐approved drugs are still used in treating filariasis but the severity of killing has declined remarkably. Several strategies with their ongoing status are in an unsatisfactory state with the success rate gradually decreasing along with the reappearance of infection giving rise to a blurred situation worldwide. In this circumstance, apoptosis could play its role as an appreciable target to combat filariasis.

Cytoskeleton disrupters like ALB block microtubule formation and reduce glucose uptake but the efficiency does not reach a satisfactory level when ALB is used in nano‐formulation the resulting antifilarial activity is augmented by the synergistic apoptotic impact. According to Zafar et al.,^168^ 100 μg/mL ALB‐CuO nanocomposite was found to be responsible for 47.3 RM value in adult *S. cervi*, while 87.1 RM value was visualized after treatment of 100 μg/mL ALB alone under dark condition but when using UV light RM value reduced surprisingly. Approximately, two times more mortality was recorded against mf when treated with ALB‐CuO nanoparticles at 100 μg/mL concentration in comparison with ALB.^168^ Moreover, azoles have some teratogenic properties thus there is always a restriction in use while most of the apoptosis‐causing agents are plant extract, so there should be no possibilities of toxic side effects, besides, they show a high efficiency as filaricide. Ethanolic extract from *C. scrabaeoides* is primarily non‐toxic to the host along with most proficient and powerful among plant extracts in targeting apoptosis since 50 μg/mL is sufficient to achieve almost 60% mortality of adult *S. cervi* as well as it has ovicidal properties.^54^ Another plant extract EEA is also non‐toxic and has an LC_50_ value of about 47.12 μg/mL against adult *S. cervi* while against mf it is 24.72 μg/mL and this report makes EEA a highly efficient killer of filarial parasites.^55^, ^134^

Ion channel blocker IVM targets GluCl channels with several successful trial reports but compared against apoptosis‐targeted agents, it has been found that IVM stands far behind in severity as filaricidal. Report from the MTT assay showed about 90% death of *W. bancrofti* mf when treated with ursolic acid at a concentration of 10 μg/mL and about 70% death when IVM was applied.^164^ Again, IVM is only successful as microfilaricidal with a little partial efficiency in adults but most of the apoptotic agents have potentiality against all the developmental stages like RSV and trans‐stilbene derivatives when treated with 50% mortality achieved at only 8.867 ± 0.82 μg/mL concentration on adult *S. cervi* but IVM never matched the level, even at 50 μg/mL concentration.^125^ Also, from another study, where 100% inhibition was reported of adult *S. cervi* after carbamo (dithioperoxo) thioate derivatives were applied, IVM can only inhibit approx. 48% at 100 μg/mL concentration.^154^ Levamisole another ion channel blocker has species‐specific microfilaricidal efficacy only against *W. bancrofti* and *B. malayi* but not in *O. volvulus* along with several histories of resistance not making a potent filaricide.^169^ Other ion channel blockers like piperazine, pyrantel, morantel and oxantel all are good in executing their action on their respective areas but are limited to their only targets like cattle, sheep, goats and dogs. In humans, these are hardly used against other nematodes, mainly hookworm, also with several limitations so there is a serious need for an apoptotic agent to eradicate human LF.

DEC is one of the most successful antifilariatic drugs that have versatile properties, that is, from blocking arachidonic metabolism, inhibiting carbohydrate metabolic process resulting in induction of oxidative stress but due to several parasite re‐emergence or resistance reports as well as due to adverse side effects, a substitute should be needed to kill parasite with the same intensity that DEC can. In this scenario apoptosis inducer, quinolone‐fused cyclic sulfonamide 8 L could be used as a substitute as well as a future antifilariatic drug since 8 L is non‐toxic to the host and secondly, it is highly active against all developmental stages of *S. cervi*. According to Mukherjee et al.,^53^ LD_50_ dose was 281.4 μM for adults, 166.9 μM for mf and to the oocyte, it was 17.3 μM and this intensity of action makes 8 L most proficient among synthetic apoptotic agents. Metabolic inhibitor suramin is also a good antifilarial drug but due to its highly toxic nature, it cannot be used frequently as it shows adverse side effects like aching of muscles and joints where all the newly discovered apoptosis targeted candidates have no harmful activity to the host.

For anti‐*Wolbachia* therapy, several antibiotics have been used for a long time to deplete the *Wolbachia*, so there should be a possibility of resistance among the antibiotics in the near future. Thus, a replacement is mandated and 8 L could help with its beneficial role in efficiency against *Wolbachia* infection.

Collectively, the aforementioned studies demonstrate that both natural and synthetic compounds capable of inducing apoptosis are highly efficient in reducing mf and adult stages of LF‐causing parasites. To further confirm the superiority of apoptogenic filaricidal agents over non‐apoptogenic compounds, a comparative study was conducted. As shown in Table 2, the majority of non‐apoptogenic filaricidal compounds exhibit lower efficacy compared to apoptosis‐inducing compounds. Moreover, the autophagy‐inducing compounds, such as Epicatechin‐3‐O‐gallate (ECG) and C‐cinnamoyl glycoside, demonstrate significantly higher LC_50_ values compared to ursolic acid, trans‐stilbene derivatives and carbamo (dithioperoxo) thioate derivatives.^113^, ^130^, ^133^, ^145^, ^158^, ^159^ Additionally, apoptosis‐inducing compounds like Tetracycline and 8 L exhibit high efficiency in depleting Wolbachia,^53^, ^114^ as indicated in Table 2.

**TABLE 2:** Comparative efficacy of apoptogenic and non‐apoptogenic filaricidal compounds.

Name of the filaricidal agent	Level of efficiency	Mode of action	References	Name of the filaricidal agent	Level of efficiency	Mode of action	References
Ursolic acid	64.6% mf mortality at 10 μg mL^−1^ and 80.1% adult mortality at 20 μg mL^−1^	Apoptosis	[130]	Epicatechin‐3‐O‐gallate (ECG)	LC_50_ of mf and adults are 4.7 and 2.2 μM, respectively	Autophagy	[170, 171]
Trans‐stilbene derivatives	LC50 of mf and adults are 9.327 ± 1.75 and 8.867 ± 0.82 μg mL^−1^, respectively	Apoptosis	[113]	Niclosamide	Express only in vitro toxicity	Autophagy	[172, 173]
Carbamo (dithioperoxo) thioate derivatives	LC50 of mf 3.89 ± 0.18 μg mL^−1^	Apoptosis	[145]	C‐cinnamoyl glycoside	LC_50_ of adults is 37.7 ± 2.1 μg mL^−1^	Autophagy	[133]
8 L (4,7‐dimethyl‐3,4,7,8‐tetrahydro‐3λ6‐[1,2]thiazino[4,3‐f]quinoline‐3,3,8‐trione)	57.6 μM can reduce *Wolbachia* by 90%	Apoptosis in *Wolbachia* symbiont	[53]	Rapamycin	Reduce *Wolbachia* by 30.7% in mf and 66% in adults	Autophagy in *Wolbachia* symbiont	[174]
Tetracycline	99% *Wolbachia* depletion	Apoptosis in *Wolbachia* symbiont	[114]	Spermidine	Reduce *Wolbachia* by 47.3% in mf and 68% in adults	Autophagy in *Wolbachia* symbiont	[174]

All the studies indicated that exploring the host's immune response will be a great strategy with no possibility of developing resistance in the near future as well as the compounds targeting the same have no toxic effect on the host and have high efficiency in killing both the mf and adult filarial parasites by triggering ROS‐induced apoptosis.

## 6 DETERMINATION OF APOPTOSIS IN FILARIAL PARASITES AND SCREENING OF ANTIFILARIAL AGENTS

Effectivity and apoptosis‐inducing properties of all the above‐mentioned antifilarial agents have been discovered experimentally in the last few years. The application of appropriate methodology for determining the induction of apoptosis has been the key to understanding the efficacy of different natural, synthetic/semi‐synthetic compounds and nanoparticles against different pathogenic filarial nematodes. Among the techniques to screening the antifilarial agents, cell viability/MTT assay and Trypan blue dye exclusion methods are the primary choices to the researchers (Table 3). Metabolic activity is one of the crucial hallmarks of the living cell.^178^ MTT (3‐(4,5‐dimethylthiazol‐2‐yl)‐2,5‐diphenyltetrazolium bromide) is a yellow tetrazolium salt and it turns into purple‐coloured formazan upon the action of the cellular mitochondrial reductase/NAD (P)H‐dependent oxidoreductase enzyme that designates cell viability.^162^ On the other side, live cells do not retain trypan blue dye while dead cell usually accumulates the dye due to loss of membrane permeability and appear as blue. Alteration in the nuclear morphology due to chromatin condensation can be easily monitored using fluorescent nuclear stains such as Hoechest, DAPI, Propidium Iodide, AO/EtBr etc. Changes in the membrane architecture specifically the position of phosphatidyl serine are a signature of cells going into apoptotic death.^179^ This could be easily diagnosed by a fluorescence stain namely FITC‐annexin‐V. All these techniques are primarily qualitative but the introduction of flow cytometry has enabled the quantitative detection of the apoptotic parameters (Table 4). For example, oocytes or mf undergoing apoptotic changes upon treatment with a drug can be very accurately examined for the quantitative induction of apoptosis and the proportion of cells in early and late‐apoptosis stages, necrotic cells and live cells can be acquired. Loss of the integrity of the genetic material is a characteristic feature of programmed cell death. Drug‐induced damage in the DNA can be efficiently determined using DNA fragmentation assay (DFA) which is a simple method to identify fragmentation of DNA in an agarose gel electropherogram.^180^ In accordance with DFA, in situ DNA fragmentation can be easily detected by TUNEL assay wherein broken double‐stranded DNA can be identified in a histological preparation of filarial parasite having exposure to an antifilarial agent.

**TABLE 3:** Screening techniques confirming the efficacy of the antifilarial agents.

Antifilarial agent screening techniques	Principle of the techniques	Detection strategy	References
MTT assay	This is a colorimetric assay, used to measure the cell metabolic activity. NAD(P)H‐dependent cellular oxidoreductase enzymes from living cells turn the yellow MTT (3‐(4,5‐dimethylthiazol‐2‐yl)‐2,5‐diphenyltetrazolium bromide) to purple formazan	Mortality percentage of treated filarial worms at the cellular level directs the efficiency of the antifilarial agents	[53, 175]
Trypan blue dye exclusion method	Trypan blue is a polar dye, impermeable to intact cell membranes but can easily enter through dead cell membranes and stain the cytoplasm of dead cells as blue	More the intensity of the blue stain will represent a greater number of worm dead cells	[55, 176]
Movability index scoring method	This visual assessment technique follows a scoring pattern on the basis of activity of the worms (extremely active as 4, moderately active as 3, a little active as 2, immovable but responding to stimuli 1 and no activity or dead as 0	Relative motility (RM) or the ratio of the motility index of treated worms to the motility index of the control group explains the filaricidal activity of the agents	[134, 177]

**TABLE 4:** Techniques determining the apoptosis in filarial parasites.

Apoptosis identifying techniques	Working principle	Specific stage of apoptosis to be determined	References
Hoechst 33342/Propidium iodide (PI) double staining	Hoechst 33342 or 2′‐[4‐ethoxyphenyl]‐5‐[4‐methyl‐1‐piperazinyl]‐2,5′‐bi‐1H‐benzimidazole trihydrochloride trihydrate is preferentially bound to the adenine‐thymine (A‐T) regions of the DNA of live or fixed cells and fluoresces at 460 nm when excited by ultraviolet (UV) light	Apoptotic nucleus reflects bright fluorescence with respect to normal as condensed forms are stained more intensely. While in necrotic cells, the edges of the nucleus display a blurry appearance. Dead cells with PI emit high‐red fluorescence.	[180]
TUNEL assay	TUNEL is a terminal deoxynucleotidyl transferase (TdT) d UTP Nick‐End Labelling technique, where the TdT enzyme labels 3′‐hydroxyl terminus of DNA breaks with biotin tagged d UTP. Subsequent mixing of streptavidin‐HRP and chromogenic substrate DAB with the labelled DNA breaks generates a brown colour that identifies apoptosis	Apoptotic nucleus appears as a brown ball with respect to a clear normal nucleus	[181]
Acridine orange/ethidium bromide (AO/EtBr) double staining	Acridine orange (AO) is a nuclear stain, permeable for both living and dead cells, whereas ethidium bromide (EtBr) stains only dead cells' nuclei. Upon intercalation with DNA, AO in normal cells emit green fluorescence and EtBr in dead cells emit orange fluorescence.	Apoptotic cells with condensed chromatin emit either bright green or orange fluorescence depending on the stage of the apoptosis, while in necrotic cells' nucleus appeared as live normal nuclei except the orange colour instead of green	[182]
DAPI staining	DAPI (4′,6‐diamidino‐2‐phenylindole) is a nuclear stain, that binds to minor grooves of adenine‐thymine regions of the DNA to emit blue fluorescence	Apoptotic cell with compromised membrane allows more DAPI within the cell, therefore, a strong blue fluorescence with observable blebbing appear	[183]
DNA ladder assay	DNA ladder assay or DNA fragmentation assay is a technique to compare the fragmentation in genomic DNA of normal cells and apoptotic cells. Here, purified DNA fragments from both types of cells resolved in Agarose gel electrophoresis and visualized under Gel‐Doc	Fragmented DNA from apoptotic cells appears as a DNA ladder	[184]
qPCR of pro‐ (CED‐3, CED‐4, Egl‐1) and antiapoptotic (CED‐9) genes	qPCR or real‐time PCR is a technique to quantify the gene of interest. Quantitative measurement of fluorescence of each cycle provides the concentration of pro‐ and anti‐apoptotic genes as Ct value	Proapoptotic genes with low Ct value and anti‐apoptotic with high commence the onset of apoptosis	[183]
Immunoblotting of pro‐ (CED‐3, CED‐4, Egl‐1) and anti‐apoptotic (CED‐9) proteins	Immunoblotting is a technique that determines the translational expression of apoptogenic proteins. Total protein extracted from the control cells and cells undergoing apoptosis are separated by SDS‐PAGE, incubated with antibodies raised against pro‐ and anti‐apoptogenic proteins, counter treatment is done with enzyme labelled IgG and visualized through enzyme‐substrate reaction or chemiluminescence	Thick dark band of apoptosis‐inducing proteins determines the onset of apoptosis	[53, 185]
Caspase Assay	Caspase assay is used to measure the activity of the apoptosis‐inducing protein Caspase3 (CED‐3 in filarid). Apoptosis inhibitor Z‐VAD‐FMK is used in this technique to compare the activity of the apoptotic proteins between normal apoptotic cells and Z‐VAD‐FMK‐treated apoptotic cells	Immunoblots of Z‐VAD‐FMK‐treated apoptotic cell appear as thin bands indicating reduced activity of the CED proteins in comparsion to untreated apoptotic cell and help in determining the onset of apoptosis	[53]
Flow cytometry	Flow cytofluorimetric analyses utilize Annexin V/Propidium Iodide (PI) double staining technique and summarize the result in a two‐dimensional dot plot. Annexin V emits a signal for detecting apoptosis while PI for necrosis and late apoptosis	Plots show double negative for Annexin V and PI denotes normal cells and necrosis for double‐positive. While positive for Annexin V and negative for PI signifies early apoptosis, negative for Annexin V and positive for PI imply late apoptosis	[186]

## 7 FUTURE DIRECTION

Natural and synthetic compounds target apoptosis. Thus, new drug candidates can impede the prevalence of filariasis. In this review article, different apoptosis‐inducing agents with the potential to eliminate filarial parasites are discussed. Since targeting apoptosis is a good approach but complete genome sequencing of *S. digitata*, a substitute model of *W. bancrofti* will help to understand the genetics behind this approach and later to develop genomic medicine that targets anti‐apoptotic mediators. However, the draft genome sequence of *S. digitata* has already been developed by Senanayaki et al.^187^ and analysis of the genetics may assist in seeking negatively regulating genes of filarial apoptosis. As we know Small interfering RNA (siRNA) silences the genes thus, the development of siRNA with complementary sequences can target anti‐apoptotic RNAs. With conventional drugs, more chemicals are used to bind with specific target proteins and show their toxic effect but compared to RNA therapeutics, nucleic acids are targeted and exert toxicity more specifically. Although there are several complications in accessing siRNA, off‐target efficacy, poor stability and delivery to targets, chemical modification such as phosphorothioate modification and nanoparticle encapsulation may overcome all such challenges.^188^ Besides siRNA, microRNA (miRNA) as well as antisense oligonucleotides (ASOs) can also be designed as future therapeutics to target genes of interest to inhibit their expression in blocking apoptosis. miRNA has an advantage in regulating the expression of multiple messenger RNA (mRNAs), so targeting miRNA could be a new way of antifilarial therapy; whereas, ASO can target miRNA safely and effectively.

Bioinformatics is flourishing in the field of drug design. With the proteomics study of *W. bancrofti* and *B. malayi*, we may find novel protein(s) that are being targeted by the apoptosis‐causing agents while metabolomics may figure out the chemical pathways which is being followed to ultimate apoptotic death. Sometimes autophagy enables apoptosis, thus activation of autophagy by inducing expression of ATG family genes may trigger apoptosis and eliminate the parasites. Knowing the novel protein and/or genes responsible for inducing apoptosis, bioinformatics can be used to design apoptotic pathways computationally. Extensive research has provided compelling evidence that filarial parasites possess immunomodulatory proteins that can effectively manipulate the host immune system.^189^, ^190^, ^191^ They can establish either proinflammatory or anti‐inflammatory milieu within the host, responsible for overt immunopathology. Taking ideas from these findings, recent studies have made significant progress in designing antifilarial immunotherapeutic agents, including vaccines and antibodies to eliminate filarial infection and restore immune homeostasis.^153^, ^192^, ^193^, ^194^, ^195^ Antifilarial potential of these future therapeutics could be investigated in the context of apoptosis and this could provide us with very insightful directions.

## 8 CONCLUSION

**FIGURE 4:**
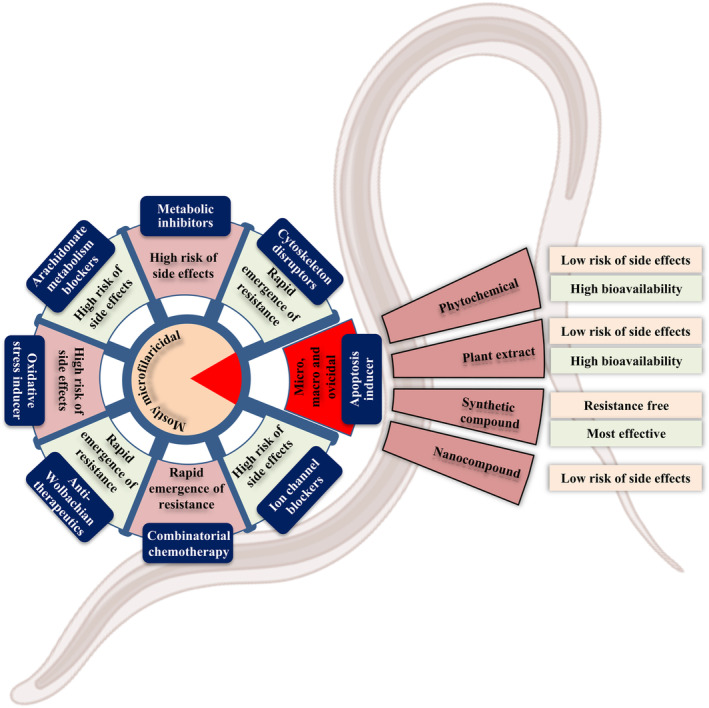
Apoptosis as an efficacious target in developing antifilarials in comparison to conventional drug targets.

In summary, analysis of all the data depicted in the available literature supports that targeting apoptosis is mostly effective than other targets for developing antifilarial drugs. Similarly, most of the available antifilarials trigger apoptotic pathways for inducing death in the lymphatic filarial parasites. Herein, Figure 4 summarizes the importance of apoptosis‐inducing natural and synthetic products in targeting the filarial parasites, including the adult parasites, emphasizing their significance over the currently available antifilarial therapeutics. In the filarial parasites, programmed cell death is an important phenomenon, typically executed by the CED pathway and the activation of the signalling molecules like CED‐9, EGL‐1, CED‐4 and CED‐3 are induced by the exposure of several chemical and/or phytocompounds.^53^, ^54^, ^134^ Therefore, targeted induction of the CED pathway is considered an effective strategy in developing antifilarial drugs and therefore apoptosis or the apoptotic pathways are considered efficacious targets for developing antifilarials. In this consequence, it can be concluded that targeted activation of apoptosis in filarial parasites through the application of natural antifilarials appears to be the way to achieve the goal of eradicating LF from endemic countries in the near future.

## AUTHOR CONTRIBUTIONS

**Nabarun Chandra Das:** Conceptualization (equal); data curation (equal); formal analysis (equal); investigation (equal); methodology (equal). **Pritha Chakraborty:** Conceptualization (equal); data curation (equal); resources (equal); validation (equal); writing – original draft (equal). **Samapika Nandy:** Writing – review and editing (equal). **Abhijit Dey:** Conceptualization (equal); writing – original draft (supporting). **Tabarak Malik:** Formal analysis (supporting); supervision (supporting); writing – review and editing (supporting). **Suprabhat Mukherjee:** Data curation (equal); methodology (equal); supervision (lead).

## CONFLICT OF INTEREST STATEMENT

The authors declare that there is no conflict of interest.

## Data Availability

Data sharing is not applicable to this article as no new data were created or analyzed in this study.
